# Calcium Transport Activity of UV/H_2_O_2_-Degraded Fucoidans and Their Structural Characterization

**DOI:** 10.3390/md22110499

**Published:** 2024-11-04

**Authors:** Biyang Zhu, Jiacheng Wang, Lijun You, Lianzhu Lin, Kuncheng Lin, Kseniya Hileuskaya

**Affiliations:** 1 School of Food Science and Engineering, South China University of Technology, Guangzhou 510640, China; fezhuby@mail.scut.edu.cn (B.Z.);; 2 Overseas Expertise Introduction Center for Discipline Innovation of Food Nutrition and Human Health (111 Center), Guangzhou 510640, China; 3 Guangdong Food Green Processing and Nutrition Regulation Technologies, Research Center, Guangzhou 510641, China; 4 Greenfresh (Fujian) Foodstuff Co., Ltd., Zhangzhou 363000, China; 5 Institute of Chemistry of New Materials, National Academy of Sciences of Belarus, 36F. Skaryna str., 220141 Minsk, Belarus

**Keywords:** calcium supplements, fucoidan, degradation, chelation, transport

## Abstract

Calcium-chelated polysaccharides have been increasingly considered as promising calcium supplements. In this study, degraded fucoidans (DFs) with different molecular weights (Mws) were prepared after UV/H_2_O_2_ treatment; their calcium-chelating capacities and intestinal absorption properties were also investigated. The results showed that the calcium-chelating capacities of DFs were improved with a decrease in Mw. This was mainly ascribed to the increased carboxyl content, which was caused by free-radical-mediated degradation. Meanwhile, the conformation of DF changed from a rod-like chain to a shorter and softer chain. The thermodynamic analysis demonstrated that DF binding to calcium was spontaneously driven by electrostatic interactions. Additionally, DF-Ca chelates with lower Mw showed favorable transport properties across a Caco-2 cell monolayer and could effectively accelerate the calcium influx through intestinal enterocytes. Furthermore, these chelates also exhibited a protective effect on the epithelial barrier by alleviating damage to tight junction proteins. These findings provide an effective free-radical-related approach for the development of polysaccharide-based calcium supplements with improved intestinal calcium transport ability.

## 1. Introduction

Calcium, as one of the vital inorganic elements in the body, plays numerous crucial physiological functions including serving as an intracellular messenger, facilitating nerve conduction, acting as an enzyme cofactor, aiding in blood clotting, and providing structural support to the skeleton and teeth [1,2]. Insufficient calcium uptake will cause bone resorption and is associated with an increased risk of rickets, osteoporosis, and hypertension [3]. Currently, the primary strategy to prevent calcium deficiency is to consume calcium-fortified products and calcium supplements, including inorganic and organic forms [4]. Among them, organic forms of calcium supplements are considered to have better absorption and bioavailability, such as calcium lactate, amino acid calcium complexes, and calcium chelates (including peptide–calcium and polysaccharide–calcium chelates) [5].

Polysaccharides have increasingly emerged as suitable alternatives to enhance calcium uptake due to their good stability, strong chelating ability, and low-cost characteristics [6,7,8]. For example, inulin and fructooligosaccharides have been found to enhance calcium bioavailability by increasing cellular calcium retention [1]. Degraded polysaccharides from *Lycium barbarum* L. leaves could increase endogenous calcium absorption in rats [9]. Fermented *Lactobacillus acidophilus* polysaccharides could improve calcium absorption and mitigate osteoporosis [8]. Though polysaccharides have been increasingly utilized to prepare calcium supplements, it is still unclear how the structural characteristics of polysaccharides affect their chelation and transport properties. For instance, the total amount of metal-ion ligands for neutral polysaccharides is relatively limited, resulting in their poor ability to chelate calcium [7]. As well, Zheng et al. (2022) reported that the intestinal absorption efficiency of polysaccharides is closely linked to their molecular weight (Mw), and high calcium-chelated content was not consistent with better calcium bioavailability [10]. Additionally, the diversity of molecular conformations in polysaccharides may have significant impacts on their chelating mode with metal ions. Yin et al. (2015) discovered that *Plantago asiatica* L. seed polysaccharides with a stiffer conformation and higher Mw were easy to chelate with calcium [11]. Ren et al. (2017) found that the calcium-chelating content of degraded polysaccharides from *Lycium barbarum* L. leaves exhibited a significant increase when their molecular chain changed from spherical conformation to linear conformation [9].

Therefore, this study aimed to prepare polysaccharide-based calcium supplements, with a particular focus on their structural changes and absorption properties. Firstly, ultraviolet (UV)/H_2_O_2_ treatment was employed to obtain the degraded fucoidan (DF) with a high carboxyl group content, thereby facilitating its chelation with calcium ions. Secondly, the changes in Mw, the carbonyl and carboxyl contents, and molecular conformations were evaluated to elucidate the chelating mechanism. Thirdly, the thermodynamic behavior for the formation of DF-Ca chelates was analyzed by isothermal titration calorimetry (ITC). In particular, the roles of different DF-Ca chelates in accelerating calcium absorption through intestinal enterocytes were also investigated. The results will pave the way for developing polysaccharide-based calcium supplements with enhanced calcium bioavailability.

## 2. Results and Discussion

### 2.1. Preparation of DF with High Carbonyl and Carboxyl Contents

**Figure 1:**
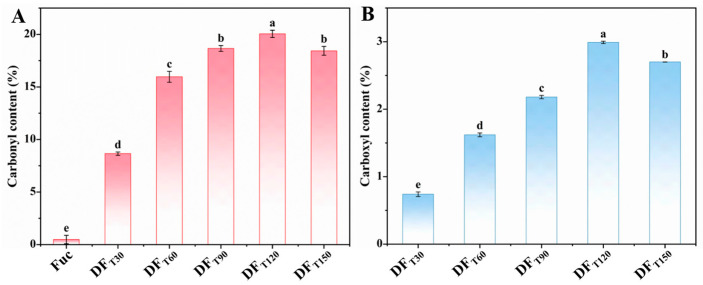
Effects of degradation time on the contents of carbonyl and carboxyl groups. (**A**) Carbonyl content. (**B**) Carboxyl content. Different letters (a–e) represent significant differences (*p* < 0.05). If two variables have different letters, they are significantly different.

As illustrated in Figure 1A, the initial carbonyl content of fucoidan (Fuc) was only 0.87%, and the fucoidan content rapidly increased to 20.04% as the degradation time reached 120 min. The rapid formation of the carbonyl group was primarily attributed to the hydrogen atom transfer reaction initiated by •OH radicals [12]. The •OH radical can selectively abstract H atoms from the C-H bonds of carbohydrates, thereby resulting in the generation of carbon-centered radicals [13]. Carbon-centered radicals rapidly reacted with O_2_ or H_2_O_2_ to form peroxyl radicals, which subsequently underwent spontaneous decomposition to obtain carbonyl products [14,15,16]. Similarly, the carboxyl content of the degraded fucoidan showed a quick increase when the degradation time was extended to 120 min (Figure 1B). The carboxyl content of the DF_T120_ sample exhibited a three-fold increase in comparison to that of the DF_T30_ sample, with values of 2.99% and 0.74%, respectively. This might be because as the degradation reaction continued, the formed carbonyl products and peroxyl radicals underwent a subsequent Baeyer–Villiger reaction to form different carboxyl products [17]. Additionally, the coupling reaction between carbon-centered radicals and •OH radicals could also result in the formation of the carboxyl group [18]. A previous study also demonstrated that neutral monosaccharides could be oxidized by •OH radical to form acidic products like hexose acids, pentose acids, tetrose acids, and glyceric acids [19]. However, when the degradation time extended to 180 min, the contents of carbonyl and carboxyl groups significantly decreased. The carboxyl group of carbohydrates was prone to undergo a decarboxylation reaction with CO_2_ and H_2_O molecules as the final products [20]. Therefore, the DF_T120_ sample was more favorable for the ionic cross-linking with calcium due to its highest contents of the negatively charged carboxyl and carbonyl groups.

### 2.2. Characterization of DF

#### 2.2.1. Molecular Weight (Mw)

**Figure 2:**
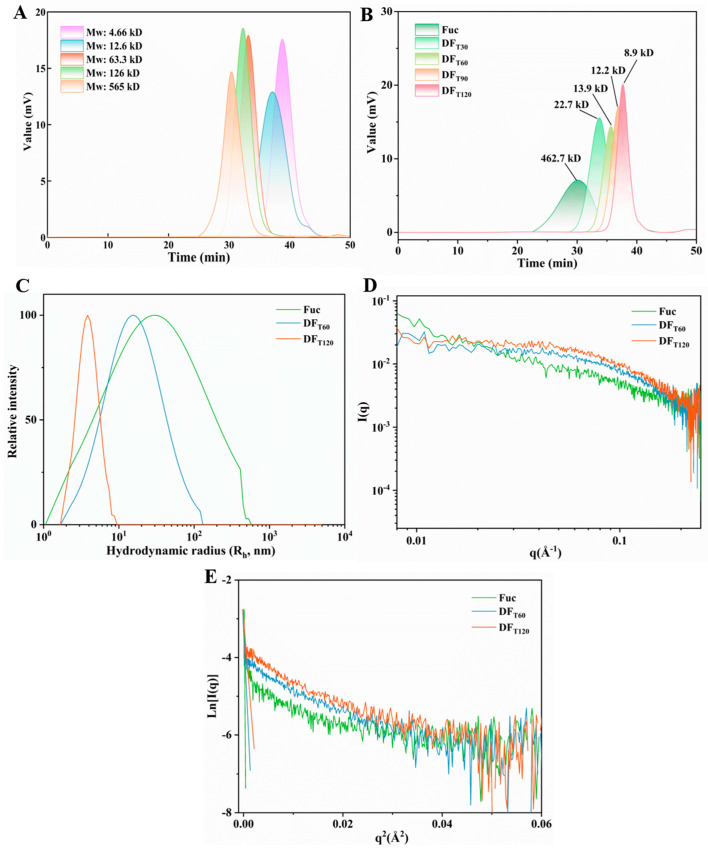
Characterization of different DFs. (**A**,**B**) Molecular weight distributions of standard dextrans and DFs. (**C**) Hydrodynamic radius (R_h_) distributions. (**D**,**E**) SAXS profiles.

Figure 2A represents the Mw distributions of standard dextrans. As shown in Figure 2B, the Mw of Fuc rapidly decreased from about 460 to 23 kDa after UV/H_2_O_2_ degradation for 30 min, suggesting that this system was highly effective in reducing the Mw of Fuc. The observed phenomenon was consistent with several previous studies, wherein the Mw of *Sargassum fusiforme* polysaccharides decreased from 289 kDa to 12.6 kDa after UV/H_2_O_2_ degradation for 2h [21]. Similarly, the Mw of *Gracilaria lemaneiformis* and modified citrus pectin polysaccharides decreased from 700 to 48.6 kDa and 524.9 to 53.3 kDa, respectively, after UV/H_2_O_2_ treatment for 30 min [22,23]. Yang et al. (2024) also reported that UV/H_2_O_2_ treatment could effectively degrade *Moringa oleifera* leaf polysaccharides [24]. Free-radical-mediated degradation would result in the cleavage of both the glycosidic bonds and pyranose rings of Fuc [25]. The Mws of DF_T60_, DF_T90_, and DF_T120_ were about 13.9, 12.2, and 8.9 kDa, respectively, showing that the decreasing rate of Mw was slow after 60 min. This indicated that the capacity of degrading Fuc by free radicals was limited in the late phase of degradation. This result could be explained by the lower levels of both Fuc and H_2_O_2_, as well as the fact that longer-chain Fuc was more easily broken down by free radicals compared to shorter-chain DF [18].

#### 2.2.2. Chain Conformation

The structural characteristics and chain conformations of polysaccharides could potentially influence their chelating manner with metal ions [7]. The molecular size distributions and SAXS profiles of different samples are depicted in Figure 2C–E, with their corresponding conformational parameters summarized in Table 1. The R_h_ values of Fuc, DF_T60_, and DF_T120_ were about 64.21, 21.07, and 5.74 nm, respectively. These results suggested that the theoretical sphere radius of Fuc exhibited a rapid decrease after UV/H_2_O_2_ treatment [26]. As shown in Figure 2D, in comparison to Fuc, the diminished scattering intensity of DF_T60_ and DF_T120_ in the low q region was attributed to their lower Mw, as evidenced by the results of Mw analysis in Section 3.2 [27]. The scattering model (I(q) ∝ q^α^) in the low q region could provide distinct conformational information of polysaccharides, including a rough interface with an α value ranging from −3 to −4, a spherical aggregation with an α value of −3, a random coil chain with an α value of −2, a swollen chain with an α value of −5/3, and a rod-like chain with an α value of −1 [28]. The α values for Fuc, DF_T60_, and DF_T120_ were fitted to be −1.06, −1.48, and −1.71, respectively, suggesting that Fuc exhibited a rod-like chain, while DF_T60_ and DF_T120_ were between a rod-like and swollen chain. Yuguchi et al. (2016) also identified a similar rod-like chain conformation of Fuc in an SAXS profile [29]. Additionally, the radius of gyration (R_g_) values were calculated by the Guinier model: ln(I(q)) = −(q^2^R_g_^2^/3), as shown in Figure 2E and Table 1. The R_g_ values of Fuc, DF_T60_, and DF_T120_ were determined as 142.36, 60.90, and 19.71 nm, respectively. The conformational parameter ρ (ρ = R_g_/R_h_) was also sensitive to the chain stiffness of polysaccharides. The polysaccharide was a sphere-like conformation for a ρ value of <0.77, a hyperbranched chain for a ρ value of 1.0–1.1, a random coil chain for a ρ value of 1.3–1.5, a flexible chain for a value of 1.5–1.8, and a rod-like chain for a ρ value > 2.0 [27,30]. The ρ value of Fuc indicated a rod-like chain conformation, in line with the α value analysis. The ρ values of DF_T60_ (ρ = 2.80) and DF_T120_ (ρ = 3.43) were higher than that of Fuc, suggesting their shorter chains resulted in a more rigid chain conformation. Ren et al. (2017) also demonstrated that the capacity of *Lycium barbarum* L. leaf polysaccharides to promote calcium absorption was associated with its conformation [9]. These results indicated that the rod-like conformation of Fuc was progressively broken down into a shorter and more swollen conformation, which might be favorable to the formation of DF-Ca chelates.

### 2.3. Calcium-Chelating Capacity of DF-Ca Chelates

**Figure 3:**
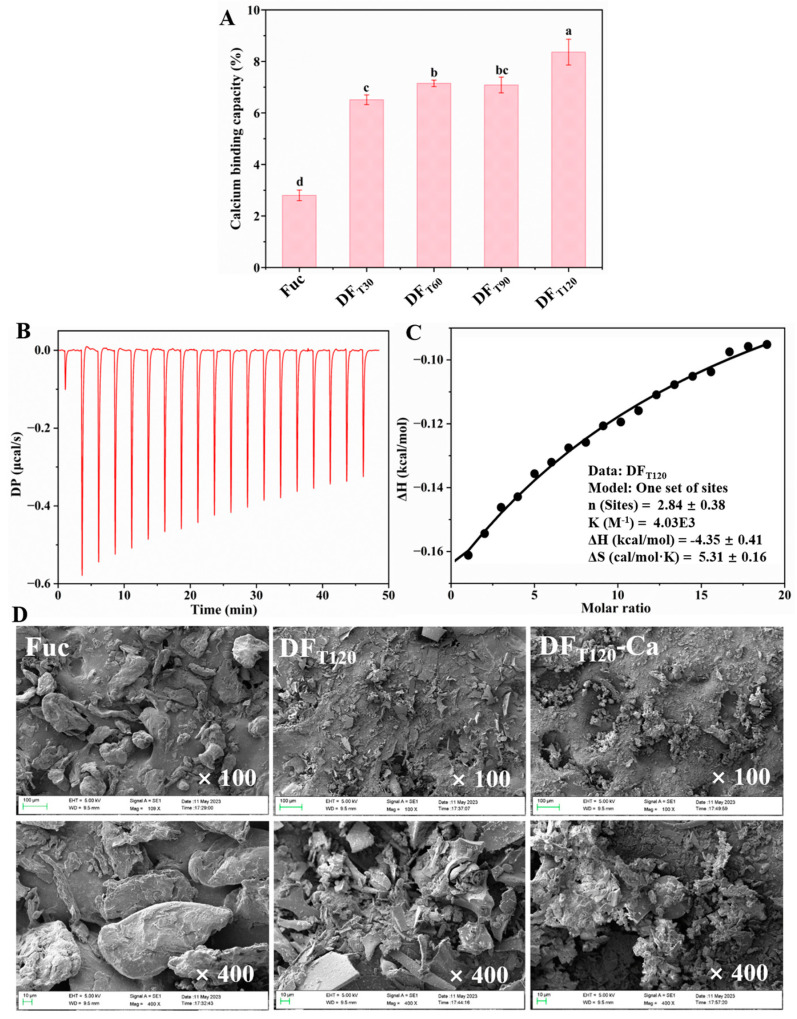
Preparation and characterization of DF-Ca chelates. (**A**) The calcium-binding capacity of different DF samples. (**B**,**C**) Thermodynamics of binding interaction between DF_T120_ and Ca^2+^. (**D**) SEM images of Fuc, DF_T120_, and DF_T120_-Ca samples.

The metal-ion-binding properties of polysaccharides strongly depended on electrostatic interactions between the metal ions and functional groups, such as hydroxyl, carboxyl, amino, and sulfate groups [31]. As shown in Figure 3A, Fuc had a lower calcium-chelating capacity (2.80%) compared to four DF samples. In Fuc, hydroxyl and sulfate groups were the main calcium-chelating ligands [32]. Chi et al. (2021) found that the sulfate group of polysaccharides exhibited a lesser affinity for binding calcium ions compared to the hydroxyl and carboxyl groups [7]. After UV/H_2_O_2_ treatment for 30 to 120 min, the calcium-chelating capacities of different DF products increased significantly, ranging from 6.51% to 8.40%. This was because the formed carbonyl and carboxyl groups in DF samples had stronger calcium-chelating capacity [7,33]. These results suggested that DF prepared by UV/H_2_O_2_ treatment was favorable for chelating with calcium.

### 2.4. Characterization of DF-Ca Chelates

#### 2.4.1. Thermodynamic Mechanism of Calcium Binding to DF

ITC has been widely employed to study the thermodynamic parameters associated with the interactions between polysaccharides and metal ions [7,34,35,36]. Figure 3B,C exhibit the calorimetric curve from the titration of CaCl_2_ into the DF_T120_ solution, as well as the associated thermodynamic data. The stoichiometry (n) of DF_T120_ and Ca^2+^ was determined to be 2.84 ± 0.38, with a binding constant (K) of 4.03 × 10^3^/mol, suggesting a relatively high binding affinity between the two molecules. The chelating reaction of DF_T120_ and Ca^2+^ could be characterized as a spontaneous exothermic process, as evidenced by the negative enthalpy change (ΔH < 0) and positive entropy change (ΔS > 0) derived from the Gibbs free energy formula. This finding was similar to the isothermal titration reaction between Ca^2+^ and exopolysaccharides, which was also attributed to the presence of carboxyl groups [36]. Qi et al. (2020) demonstrated that negative enthalpy change and positive entropy change during the binding of calcium to polysaccharides were caused by electrostatic interaction [34]. These results indicated that the binding reaction between DF_T120_ and Ca^2+^ was spontaneously driven by electrostatic binding interactions, which were sensitive to the ionic environment.

#### 2.4.2. Microstructural Analysis

The surface topographies of Fuc, DF_T120_, and DF_T120_-Ca were also analyzed and are shown in Figure 3D. The undegraded Fuc exhibited a relatively aggregated and irregular spherical morphology, a characteristic attributed to its high Mw and aggregating behavior. After UV/H_2_O_2_ treatment, the surface of DF_T120_ showed a large amount of smooth and thin slices. This alteration was also observed in *Sargassum fusiforme* polysaccharides treated with a UV/H_2_O_2_ system [37]. The morphological change in DF_T120_ might be attributed to the cleavage of the glycosidic bonds by degradation, which reduced the intermolecular cross-linking degree and facilitated the depolymerization of the polysaccharide chains, thus creating more calcium-binding sites. Furthermore, compared to DF_T120_, DF_T120_-Ca showed a higher quantity of fragmented and compact fragments containing internal cavities.

### 2.5. Transport Capacities of Different DFs Across Caco-2 Cell Monolayers

**Figure 4:**
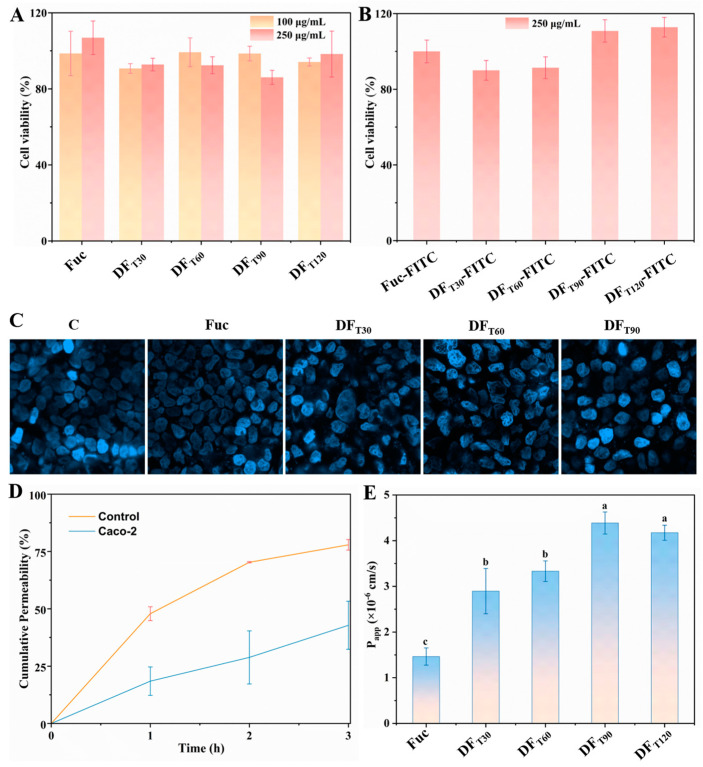
Transport capacities of different DFs and DF-FITC samples across Caco-2 cell monolayers. (**A**,**B**) Effects of DFs and DF-FITC on the cell viability, respectively. (**C**) DAPI staining of the cell nucleus. (**D**) The permeability of standard sodium fluorescein. (**E**) The P_app_ values of different samples from AP to BL side.

Caco-2 cells could slowly differentiate to form cell monolayers that had similar morphological and biochemical features to the small intestinal villus epithelium. Therefore, Caco-2 cell monolayers have been widely used for studying the intestinal transport capacities of nutrients [10]. The cytotoxic effects of DF and DF-FITC samples on Caco-2 cells are displayed in Figure 4A,B. The viability of Caco-2 cells was not significantly changed by DF and DF-FITC samples at 100 and 250 μg/mL. The results showed that all tested samples before and after fluorescent labeling were non-toxic and could be used in subsequent experiments. The observation of the cell nucleus revealed that Caco-2 cells formed a cohesive monolayer structure (Figure 4C). The compactness of the monolayer was further verified by the evaluation of the permeability using standard sodium fluorescein, as shown in Figure 4D. Compared with the control plate, the cumulative permeability of sodium fluorescein across Caco-2 cell plates exhibited a significant decrease, thereby confirming the successful establishment of the Caco-2 cells monolayer model, according to previous findings [38].

The transport pathways of polysaccharides across the intestine epithelial monolayers mainly involved the paracellular, transcellular, and M-cell-mediated pathways [10]. The transport of polysaccharides through the paracellular and transcellular pathways was mediated by tight junctions and transcytosis, respectively. Previous studies reported that Fuc was absorbed by clathrin-mediated endocytosis in Caco-2 cell monolayers, a recognized transcellular pathway [10,39]. According to a widely accepted standard, pharmaceutical compounds with good, moderate, and poor absorption rates should exhibit P_app_ values > 1 × 10^−5^ cm/s, between 1 × 10^−5^ and 0^−6^ cm/s, and <1 × 10^−6^ cm/s, respectively [40]. As shown in Figure 4E, the P_app_ value of Fuc was 1.46 × 10^−6^ cm/s, suggesting moderate absorption of Fuc in the small intestine. The P_app_ value of DF samples significantly increased with the increase in UV/H_2_O_2_ degradation time. The P_app_ value of DF_T120_ reaching 4.39 × 10^−6^ cm/s indicated a better absorption property. It has been reported that the intestinal transport capability of polysaccharides is influenced by many factors, including charge, Mw, chain conformation, and dosage [10]. Therefore, UV/H_2_O_2_ treatment could improve the intestinal absorption rate of DF samples because it led to an increase in the total charge, a decrease in Mw, and a shorter and more flexible chain conformation.

### 2.6. Changes in Intracellular Calcium Level by DF-Ca Chelates

**Figure 5:**
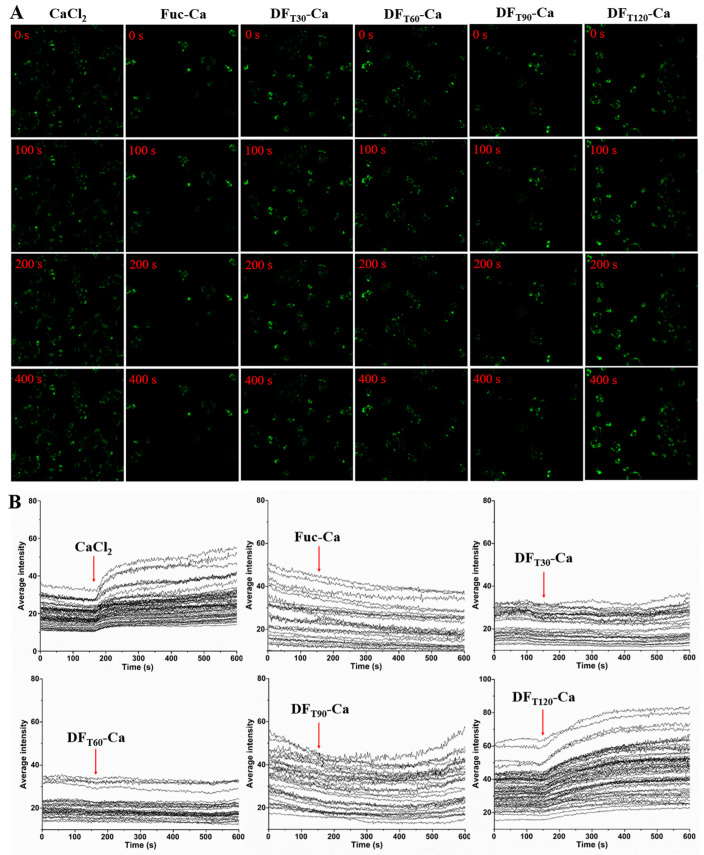
Effects of different Fuc-Ca and DF-Ca samples on the intracellular calcium level of Caco-2 cells. (**A**) Representative CLSM images at 0, 100, 200, and 400 s, with an image scale of 638.9 μm × 638.9 μm and magnification of 20×. (**B**) Changes in the intracellular calcium level.

To identify the capacities of different DF-Ca chelates in facilitating calcium transport, the changes in intracellular calcium levels were monitored by CLSM, as shown in Figure 5. After stimulation with CaCl_2_, the fluorescence intensity exhibited a rapid and high increase, confirming that calcium salt could be quickly absorbed by Caco-2 cells. However, it was reported that calcium salt tended to form insoluble salt precipitation in the weakly alkaline intestinal tract, thereby causing some intestinal side effects such as flatulence and indigestion [41,42]. The fluorescence intensity in cells was minimally affected by the presence of Fuc-Ca, DF_T30_-Ca, and DF_T60_-Ca. This phenomenon was attributed to the poor cellular absorption of Fuc, DF_T30_, and DF_T60_ when the Mw was higher than 14 kDa, as explained in Section 3.2 and Section 3.5. Changes in the Mw of polysaccharides had crucial implications for both their uptake rates and absorption mechanisms [10]. DF_T90_-Ca treatment slightly raised the intracellular calcium level within 5 min, while DF_T120_-Ca treatment quickly showed a significant absorption signal. Therefore, compared with Fuc-Ca, DF_T120_-Ca could effectively promote calcium influx via the intestinal epithelial membrane. These results demonstrated that DF displayed an enhanced absorption property when its Mw was lower due to UV/H_2_O_2_ degradation.

### 2.7. Influence of Different DF-Ca Chelates on Caco-2 Cell Barrier

**Figure 6:**
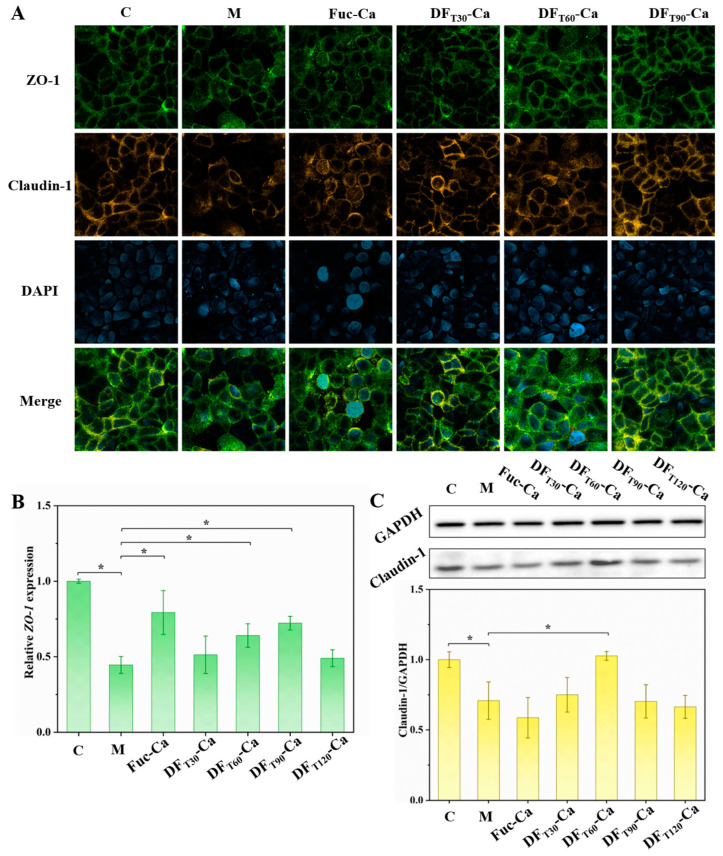
Effects of different DF-Ca chelates on gene transcription and protein expression levels related to the intestinal barrier. (**A**) Representative immunofluorescence images. (**B**) The transcription level of the ZO-1 gene. (**C**) The expression of the claudin-1 protein.

As a potential polysaccharide-based calcium supplement, DF not only should have a good ability to promote calcium absorption, but also requires no negative effects on the intestinal barrier. Therefore, the effects of different DF-Ca chelates on the gastrointestinal barrier were also studied. The gastrointestinal barrier is composed of intercellular tight junction (TJ) proteins situated in the apical region of epithelial cells [43]. Among the TJ proteins, ZO-1 and claudin-1 are two important transmembrane proteins within the epithelial barrier [44]. The protective effect of DF-Ca on LPS-induced damage to TJ proteins was evaluated by immunofluorescence staining of ZO-1 and claudin-1. As shown in Figure 6A, in the control group, ZO-1 and claudin-1 proteins exhibited a smooth and uninterrupted circular distribution at the junctions of Caco-2 cells. However, the expression levels of ZO-1 and claudin-1 proteins in the model group were significantly decreased compared with those in the control group, demonstrating the detrimental effect of LPS on TJ proteins in the intestinal epithelial barrier [45]. After DF_T30_-Ca, DF_T60_-Ca, and DF_T90_-Ca treatment, green and yellow fluorescence intensities became significantly stronger and were fully restored to an uninterrupted circular pattern. In addition, the relative transcriptional level of the *ZO-1* gene and the expression level of the claudin-1 protein were also investigated. As shown in Figure 6B,C, the *ZO-1* transcription and claudin-1 expression significantly decreased compared with those in the control group, which corroborated the change in fluorescence intensities. Moreover, the transcription of the *ZO-1* gene exhibited a significant increase following treatments with DF_T60_-Ca and DF_T90_-Ca, while the expression of the claudin-1 protein also significantly increased after DF_T60_-Ca treatment. These findings suggested that DF_T60_-Ca treatment might effectively prevent damage to the intestinal epithelial barrier. Chen et al. (2021) also found that degraded polysaccharides from *Sargassum fusiforme* (PSF-T2) treated by UV/H_2_O_2_ could inhibit intestinal epithelial barrier damage in RAW 264.7 cells and a BABL/c mouse model [46].

## 3. Materials and Methods

### 3.1. Materials and Reagents

Fucoidan, catalase (≥200 kU), FITC, and sodium fluorescein were purchased from Aladdin Biochemical Co., Ltd. (Shanghai, China). Hydroxylamine chloride, glyoxal, tyramine, NaBH_3_CN, and 4% paraformaldehyde were purchased from Macklin Biochemical Co., Ltd. (Shanghai, China). Dulbecco’s Modified Eagle’s Medium (DMEM) and fetal bovine serum (FBS) were purchased from Gibco Biotechnology Co., Ltd. (Grand Island, NY, USA). An MTT kit was purchased from Jiancheng Bioengineering Co., Ltd. (Nanjing, China). DAPI and Trizol were purchased from Sigma-Aldrich Co., Ltd. (Carlsbad, CA, USA). Fluo-4/AM, Pluronic F-127, rabbit anti-Zonula occludes-1 (ZO-1) and anti-claudin-1 primary antibodies, FITC-conjugated and Cy3-conjugated goat secondary antibodies, RIPA lysis buffer, and enhanced chemiluminescence (ECL) reagent were purchased from Beyotime Co., Ltd. (Shanghai, China). A horseradish-peroxidase-conjugated secondary antibody was purchased from Servicebio Co., Ltd. (Wuhan, China). RevertAid First Strand cDNA Synthesis Kit, PowerUp^TM^ SYBR^TM^ Green Mix, PVDF, and TBST were purchased from Thermo Fisher Scientific Co., Ltd. (Shanghai, China).

### 3.2. Degradation of Fucoidan Using UV/H_2_O_2_ System

The degradation of fucoidan was conducted in a photoreaction device, equipped with a UV lamp (HOPE-MED 8140, λ_max_ = 313 nm, Tianjin Hepu Company, Tianjin, China), according to our previous study [18,21]. Briefly, to produce the degraded fucoidan with the highest carboxyl content, a solution consisting of 2.5 mg/mL fucoidan and 150 mM H_2_O_2_ (30% *v*/*v*) was exposed to UV degradation for varying durations at a radiation intensity of 1830 μW/cm^2^. After the reaction, an appropriate amount of catalase was added to the mixed solution to eliminate excess H_2_O_2_ for 0.5 h. The reaction solution was concentrated by a 55 °C rotary evaporator (Heidoph, Nuremberg, Germany) and subsequently subjected to a dialysis membrane (Mw cut-off 0.5 kDa) for 48 h. The final solutions were freeze-dried by a vacuum lyophilizer (Alpha 1-2 LD plus, Christ, Germany) for 48 h to obtain different degraded fucoidan samples, named Fuc, DF_T30_, DF_T60_, DF_T90_, DF_T120_, and DF_T150_, referring to different treatment times, 0, 30, 60, 90, 120, and 150 min, respectively.

### 3.3. Contents of Carbonyl and Carboxyl Groups

The carbonyl contents of different degraded fucoidan samples were quantified using a previous method with slight modifications [47]. Briefly, 2 mL of 0.5 mg/mL freeze-dried sample solution was combined with 2 mL of 0.5% hydroxylamine chloride solution and 2 mL of 2% sodium acetate solution. Subsequently, the mixtures were subjected to incubation at 50 °C for 15 min. The absorbances of the mixtures were quantified at a wavelength of 233 nm by a UV765 spectrophotometer (Yoke Instrument, Shanghai, China). Standard glyoxal solutions (0–1 mM) were used to obtain the calibration curve of carbonyl content. The carboxyl contents of different samples were assessed according to a previous method with slight modifications [48]. Initially, 10 mg of each sample was dissolved in 10 mL of 0.05 M calcium acetate solution. The sample solution was then titrated using a 0.01 M NaOH solution until its pH reached 8.2. Furthermore, a blank Fuc group consisting of 10 mL of 0.05 M calcium acetate solution was also titrated. The carboxyl content was calculated from the volume of NaOH solution utilized.

### 3.4. Characterization of Degraded Fucoidan

#### 3.4.1. Molecular Weight (Mw) Distributions

The Mw of different samples was determined by high-performance gel permeation chromatography (HPGPC) with a refractive index detector (Model 2414, Waters Co., Milford, MA, USA) [21]. Two aqueous columns (TSK-GEL G-6000 PWXL column and G-3000PWXL column, 7.8 × 300 mm i.d., Tosoh Co, Tokyo, Japan) were employed in the experiment. The sample solution (2 mg/mL) was filtered through a 0.22 μm membrane. The mobile phase consisting of 20 mM KH_2_PO_4_ was pumped at a flow rate of 0.5 mL/min, with an injection volume of 20 μL. Dextran standards with different Mws (4.66, 12.6, 63.3, 126, and 565 kDa) were used as the references.

#### 3.4.2. Dynamic Light Scattering (DLS)

The hydrodynamic parameters were studied using a BI-200SM DLS/SLS light scattering goniometer (Brookhaven Instruments, New York, USA) at 25 °C, equipped with a 632.8 nm He-Ne laser and a multiple digital time correlator. The samples were all dissolved in 0.1 M NaCl solution to a concentration of 0.2 mg/mL. The hydrodynamic radius (R_h_) was calculated from the Stoke–Einstein equation:

(1) $$Rh=\frac{kBT}{6\pi \eta D0}$$

The equation includes the Boltzmann constant (k_B_), the absolute temperature (T), the solvent viscosity (η), and the translational diffusion coefficient (D_0_).

#### 3.4.3. Small-Angle X-Ray Scattering (SAXS)

The conformational changes of samples were studied by SAXS using a Xenocs Xeuss 2.0 instrument (XENOCS, Sassenage, France), equipped with an Eiger2R 1M detector. The scattering intensity was measured within the range of 0.008–0.26 Å^−1^ under vacuum conditions. The samples were dissolved in 0.1 M NaCl solution to a concentration of 15 mg/mL. The final SAXS profiles were obtained by subtracting the background signal from 0.1 M NaCl solution.

### 3.5. Preparation of Degraded Fucoidan–Calcium (DF-Ca) Chelates

Briefly, the DF sample and CaCl_2_ were dissolved in 10 mM Tris-HCl buffer (pH = 8.0) at a concentration of 10 mg/mL. Equal volumes of polysaccharide solution and CaCl_2_ solution were mixed and then stirred in a water bath (C-MAG HS4, IKA Instrument Co., Ltd., Staufen im Breisgau, Germany) at 30 °C for 4 h, respectively. Then, the solutions were mixed with six times the volume of absolute ethanol and centrifuged twice at 8000 rpm for 10 min. The collected precipitates were redissolved in 10 mL of ultrapure water and subjected to a dialysis membrane (Mw cut-off 0.5 kDa) for 48 h. The final solutions were then freeze-dried for 48 h to obtain DF-Ca chelate. The calcium-binding contents of different chelates were measured using a flame atomic absorption spectrometer (240FS AA, Agilent Technologies, Santa Clara, CA, USA) according to a previous study [42]. Similarly, different DF-Ca chelates were prepared by chelating different DFs (Fuc, DF_T30_, DF_T60_, DF_T90_, and DF_T120_) with calcium for 4 h, designated as Fuc-Ca, DF_T30_-Ca, DF_T60_-Ca, DF_T90_-Ca, and DF_T120_-Ca, respectively.

### 3.6. Characterization of DF-Ca Chelates

#### 3.6.1. Isothermal Titration Calorimetry (ITC)

A Microcal PEAQ-ITC calorimeter (Malvern Panalytical, Malvern, UK) was employed to measure the thermodynamic parameters between calcium ions and DF_T120_. The CaCl_2_ and DF_T120_ were both dissolved in 10 mM Tris-HCl buffer (pH 8.0). The CaCl_2_ solution (10 mM) was sequentially injected into the DF_T120_ solution (2.5 mg/mL), using 10 mM Tris-HCl buffer as the blank. Each injection had a duration of 20 s, with an interval of 150 s between successive injections. The solution in the sample cell was maintained at 25.0 ± 0.1 °C and stirred at 400 rpm. The stoichiometry (n), binding constant (K), enthalpy (ΔH), and entropy change (ΔS) were calculated.

#### 3.6.2. Scanning Electron Microscopy (SEM)

The surface morphologies of Fuc and DF samples were analyzed using an EVO 18 SEM at an accelerating voltage of 5 kV (Carl Zeiss Microscopy GmbH, Oberkochen, Germany). The samples were affixed to a double-sided conductive metal tape and sprayed with gold under vacuum. Microscopic images were captured at magnifications of 100 × and 400 ×.

### 3.7. Transport Studies of Different DFs Across Caco-2 Cell Monolayers

#### 3.7.1. Fluorescent Labeling of DF

Fuc and DF samples were fluorescently labeled according to previously described methods [38]. Briefly, 400 mg of samples, 400 mg of tyramine, and 150 mg of NaBH_3_CN were all dissolved in 15 mL of 0.2 M PBS (pH 8.0). The mixture was kept in a 37 °C water bath for 96 h. The solutions were centrifuged at 10,000 rpm for 10 min and then dialyzed by a dialysis membrane (Mw cut-off 0.3 kDa) for 96 h. Further, 30 mg of FITC was added to the solutions and adjusted to pH 8.5 using 0.5 M NaHCO_3_ solution in the dark for 24 h. The final solutions were precipitated with seven times the volume of absolute ethanol and centrifuged at 10,000 rpm for 10 min. The precipitations were freeze-dried for 48 h to obtain different fluorescent labeling samples, named Fuc-FITC, DF_T30_-FITC, DF_T60_-FITC, DF_T90_-FITC, and DF_T120_-FITC, respectively. The fluorescence intensities of various sample solutions (12.5 to 250 μg/mL) were measured by a confocal laser scanning microscope (CLSM) (LSM 900, Carl Zeiss Microscopy GmbH, Oberkochen, Germany) to obtain the standard curves, at excitation and emission wavelengths of 490 and 520 nm, respectively.

#### 3.7.2. Cell Cultivation and Viability

Caco-2 cells were cultured in DMEM supplemented with 10% FBS in a 5% CO_2_ atmosphere at 37 °C. The cells were seeded in a 96-well plate at the density of 1 × 10^4^ cells/well until the confluence reached approximately 80%. Then, 100 μL of DF and DF-FITC samples (100 or 250 μg/mL) were introduced into the 96-well plate. Following an incubation period of 24 h, the cultured medium was discarded. The commercial MTT kit was used to determine cell viability according to the provided guidelines.

#### 3.7.3. Transport Capacity of DF-FITC Across Caco-2 Cell Monolayers

The Caco-2 cells were seeded on 12-well polycarbonate transwell plates at a density of 2 × 10^5^ cells/mL in the apical chamber (AP) with 0.5 mL of complete medium, while 1.5 mL of cell-free complete medium was introduced into the basolateral chamber (BL). The medium was replaced every two days during the first week and changed daily in the subsequent weeks. The Caco-2 cell monolayers exhibiting a transepithelial electrical resistance (TEER) value (Millicell-ERS, Millipore, Billerica, MA, USA) above 500 Ω/cm^2^ were selected for the transmembrane transport studies. Briefly, 50 µL of sodium fluorescein (100 µg/mL) was added to the AP side. Then, 50 µl of the medium at the BL side was withdrawn every 1 h to determine the membrane integrity of the cell monolayers [39]. Additionally, the Caco-2 cell monolayers were separated from the polycarbonate plate and treated with 4% paraformaldehyde. Subsequently, the cell nucleus was stained with DAPI and observed using a CLSM.

After the establishment of the Caco-2 cell monolayers, the medium was removed, and the cells were preincubated with HBSS solution (pH = 7.4) at 37 °C for 30 min. The HBSS solution was then removed from the AP side and replaced with 0.5 mL of 250 μg/mL Fuc-FITC or DF-FITC solution (dissolved in HBSS), with 1.5 mL of fresh HBSS added in the BL side. At 15, 30, 60, 90, 120, 150, and 180 min, a certain volume of solution was withdrawn from the BL side and replaced with an equal volume of fresh HBSS solution. The fluorescence intensities of the collected samples were detected to calculate the apparent permeability coefficient (P_app_) values by the following equation [40]:

(2) $$P\mathrm{app}=\frac{\mathrm{dQ}/\mathrm{dt}}{A\times \;C0}$$

The equation includes the cumulative transport volume per unit time (dQ/dt), the membrane surface area (A, cm^2^), and the initial concentration (C_0_, μg/mL).

### 3.8. Calcium Transport-Promoting Activity of DF-Ca Chelate

#### 3.8.1. Effects of DF-Ca Chelates on Intracellular Calcium Level

The increment in intracellular calcium carried by DF-Ca chelates was monitored by our previous method with some modifications [41]. Caco-2 cells were seeded in plastic Petri plates (35 mm diameter, Sorfa, Deqing, China) at the density of 5 × 10^5^ cells/plate for 24 h. The cells were washed with HBSS solution (pH = 7.4) and incubated with 10 μM Fluo-4/AM fluorescence indicator (dissolved in 20% Pluronic F-127 solution) for 30 min. Furthermore, the extracellular Fluo-4/AM indicator was rinsed with HBSS solution three times, and the plates were placed on a CLSM. The cells were then treated with different sample solutions (containing 10 mM Ca^2+^), and the resulting fluorescence intensity of each cell was recorded at excitation and emission wavelengths of 484 and 525 nm, respectively.

#### 3.8.2. Effects of DF-Ca on Caco-2 Cell Barrier

(1) Immunofluorescence staining

Caco-2 cells were cultured in 12-well plates at a seeding density of 2 × 10^5^ cells/well until reaching a cell confluence of approximately 80%. Following pretreatment with 1 mL of LPS solution (1 μg/mL) for 24 h, 1 mL of Fuc or DF-Ca solution (250 μg/mL) was then added for an additional 24 h. The cells were fixed with 4% paraformaldehyde and blocked with 2% BSA for 1 h. ZO-1 and claudin-1 proteins were immunostained with rabbit anti-ZO-1 and anti-claudin-1 primary antibodies overnight and then incubated with FITC-conjugated and Cy3-conjugated goat secondary antibodies for 50 min. After being rinsed with PBS three times, the cell nucleus was stained with DAPI. All fluorescence images were also obtained using a CLSM.

(2) qRT-PCR analysis

The mRNA level of *ZO-1* was analyzed by quantitative RT-PCR (qRT-PCR). The total mRNA of Caco-2 cells in 12-well plates was isolated by Trizol reagent. cDNA was synthesized by the RevertAid First Strand cDNA Synthesis Kit according to the manufacturer’s instructions. The qRT-PCR amplification process of *ZO-1* was conducted by a Bio-Rad system (Bio-Rad Laboratories, Inc., Hercules, CA, USA) using PowerUp^TM^ SYBR^TM^ Green Mix. The normalization of *ZO-1* gene expression value was conducted with the housekeeping gene glyceraldehyde-3-phosphate dehydrogenase (GAPDH) as a reference.

(3) Western blot analysis

Total protein was extracted from Caco-2 cells using RIPA lysis buffer. The collected protein was separated by 10% sodium dodecyl sulfate–polyacrylamide gel electrophoresis (SDS-PAGE) and then transferred onto a PVDF membrane. After blocking with 5% non-fat milk in TBST buffer for 2 h at room temperature, the membrane was incubated with the anti-claudin-1 primary antibody overnight. It was then washed with TBST three times and exposed to the horseradish-peroxidase-conjugated secondary antibody for 1 h. Signals were visualized using Plus ECL reagent.

### 3.9. Statistical Analysis

Each experiment was conducted at least three times independently. The data were expressed as the mean ± SD values and analyzed with one-way analysis of variance (ANOVA) following Tukey’s multiple comparison tests using the SPSS 26.0.0.0 software (SPSS Inc., Chicago, IL, USA). The statistical significance was set as the level of *p* < 0.05.

## 4. Conclusions

Degraded fucoidan (DF)–calcium chelates were prepared in this study, and their absorption properties were also investigated. After UV/H_2_O_2_ treatment, DF showed an increased carboxyl content and an enhanced calcium-chelating ability. This was closely associated with a decrease in Mw and a conformational change from the rod-like chain to the more flexible short-chain structure, caused by free-radical-mediated degradation. The binding of DF with Ca^2+^ was dominated by favorable enthalpic effects (ΔH of −4.35 kcal/mol) reflecting electrostatic interactions. Moreover, in a Caco-2 cell monolayer model, DF-Ca chelates with lower Mw exhibited better absorption properties across the intestinal epithelial barrier and could effectively accelerate the calcium influx. Additionally, DF-Ca also showed a protective effect on intercellular junctions by regulating the gene transcription and protein expression levels of ZO-1 and claudin-1. This work provides a promising UV/H_2_O_2_ degradation strategy for developing polysaccharide-based calcium supplements with excellent calcium-chelating and intestinal absorption properties.

## Figures and Tables

**Table 1:** Conformational parameters of Fuc and DF samples.

Samples	α value	R_h_ (nm)	R_g_ (nm)	R_g_/R_h_
Fuc	−1.06 ± 0.03	64.21 ± 1.69	142.36 ± 13.65	2.22
DF_T60_	−1.48 ± 0.02	21.07 ± 0.77	60.90 ± 11.50	2.80
DF_T120_	−1.71 ± 0.03	5.74 ± 0.09	19.71 ± 1.64	3.43

## Data Availability

The original contributions presented in this study are included in the article. Further inquiries can be directed to the corresponding author.
